# Deep Learning for Automatic Differentiation of Mucinous versus Non-Mucinous Pancreatic Cystic Lesions: A Pilot Study

**DOI:** 10.3390/diagnostics12092041

**Published:** 2022-08-24

**Authors:** Filipe Vilas-Boas, Tiago Ribeiro, João Afonso, Hélder Cardoso, Susana Lopes, Pedro Moutinho-Ribeiro, João Ferreira, Miguel Mascarenhas-Saraiva, Guilherme Macedo

**Affiliations:** 1 Department of Gastroenterology, São João University Hospital, Alameda Professor Hernâni Monteiro, 4200-427 Porto, Portugal; 2 World Gastroenterology Organisation Gastroenterology and Hepatology Training Center, 4200-427 Porto, Portugal; 3 Faculty of Medicine of the University of Porto, Alameda Professor Hernâni Monteiro, 4200-427 Porto, Portugal; 4 Department of Mechanical Engineering, Faculty of Engineering of the University of Porto, Rua Dr. Roberto Frias, 4200-465 Porto, Portugal; 5 INEGI—Institute of Science and Innovation in Mechanical and Industrial Engineering, Rua Dr. Roberto Frias, 4200-465 Porto, Portugal

**Keywords:** pancreatic cystic lesions, mucinous cystic neoplasm, intraductal papillary mucinous neoplasm, endoscopic ultrasound, artificial intelligence

## Abstract

Endoscopic ultrasound (EUS) morphology can aid in the discrimination between mucinous and non-mucinous pancreatic cystic lesions (PCLs) but has several limitations that can be overcome by artificial intelligence. We developed a convolutional neural network (CNN) algorithm for the automatic diagnosis of mucinous PCLs. Images retrieved from videos of EUS examinations for PCL characterization were used for the development, training, and validation of a CNN for mucinous cyst diagnosis. The performance of the CNN was measured calculating the area under the receiving operator characteristic curve (AUC), sensitivity, specificity, and positive and negative predictive values. A total of 5505 images from 28 pancreatic cysts were used (3725 from mucinous lesions and 1780 from non-mucinous cysts). The model had an overall accuracy of 98.5%, sensitivity of 98.3%, specificity of 98.9% and AUC of 1. The image processing speed of the CNN was 7.2 ms per frame. We developed a deep learning algorithm that differentiated mucinous and non-mucinous cysts with high accuracy. The present CNN may constitute an important tool to help risk stratify PCLs.

## 1. Introduction

Pancreatic cystic lesions (PCLs) are very common. A recent systematic review including 17 studies found a pooled prevalence of 8% [1]. PCLs include a wide range of entities, namely congenital, inflammatory, and neoplastic lesions. Patients with PCLs have an increased risk of pancreatic malignancy compared with the general population [2]. However, malignancy occurs virtually only in those with PCLs of mucinous phenotype. Intraductal papillary mucinous neoplasm (IPMN) is the most common pancreatic cystic neoplasia, accounting for nearly half of pancreatic resections due to cystic lesions at a reference academic hospital in the USA [3].

The diagnosis of PCLs based on endoscopic ultrasound (EUS) has important limitations [4]. In fact, the range of accuracy in differentiating mucinous from non-mucinous lesions is 48–94% with a sensitivity of 36–91% and a specificity of 45–81% [4]. However, one of the main limitations of EUS is its low interobserver agreement for the diagnosis of neoplastic versus non-neoplastic lesions and the determination of the specific type of PCL. These concerns remain valid for a wide spectrum of endoscopists, with different degrees of expertise in EUS (experts, semi-experts, or novices) [5,6].

The application of artificial intelligence (AI) algorithms for the interpretation of medical imaging has been the focus of intense research across several areas [7,8]. The implementation of these automated systems for the automatic analysis of endoscopic images has provided promising results [9]. The ever-increasing computational power allows the analysis of large image datasets through deep learning algorithms. Convolutional neural networks (CNNs) are a type of multi-layer deep learning algorithm resembling the visual cortex, which is tailored for automatic image analysis [10].

To date, only a small number of studies reported the use of deep learning systems for the automatic interpretation of EUS images [11]. To optimize the diagnosis based on EUS morphology and mitigate the low interobserver agreement, we aimed to develop a CNN algorithm for the automatic diagnosis of mucinous PCLs using EUS images.

## 2. Materials and Methods

### 2.1. Patient Population and Study Design

We conducted a retrospective study using a prospectively maintained hospital database of patients submitted to EUS for PCL characterization. All patients whose EUS exam was recorded as a video file were included. All videos were recorded using the same EUS device. Images retrieved from these examinations were used for the development, training, and validation of a CNN-based model for the automatic identification of mucinous PCLs. 

This study was approved by the ethics committee of São João University Hospital/Faculty of Medicine of the University of Porto (CE 41/2021) and was conducted respecting the Declaration of Helsinki. This study is of a non-interventional nature.

### 2.2. Data Collection

We retrieved the videos from 28 patients for high-quality EUS image analysis. These images comprised still frames acquired during the EUS procedure as well as images obtained through the decomposition of recorded videos into frames. The fragmentation of videos into still images was performed using the VLC media player (VideoLAN, Paris, France). The complete set of images was evaluated by an expert in EUS (FVB) with an experience of more than 1000 EUS exams. All non-relevant frames were excluded. A total of 5505 images were ultimately extracted. From this pool, 3725 depicted mucinous PCLs and 1780 showed non-mucinous PCLs.

Clinical and demographic data were obtained from the electronic clinical record of each patient. Any information deemed to potentially identify the subjects was omitted. Each patient was assigned a random number in order to guarantee effective data anonymization. A team with Data Protection Officer (DPO) certification confirmed the non-traceability of data and conformity with the general data protection regulation (GDPR).

### 2.3. Endoscopic Ultrasound Procedures and Definitions

All EUS procedures were performed under anesthesiologist-directed sedation using linear echoendoscopes (Olympus^®^ GF-UCT180 and Olympus^®^ GF-UC140) coupled with an Olympus^®^ EU-ME2 ultrasound processor under anesthesiologist-directed sedation. Cyst type was determined based on surgical specimen, intracystic biopsy forceps samples (Moray^®^ micro forceps, STERIS) or cyst fluid cytology combined with carcinoembryonic antigen (CEA) and glucose fluid levels. PCLs were considered mucinous if cytology revealed mucinous epithelial cells or, in their absence, CEA fluid levels >192 ng/mL and glucose levels <50 mg/dL. Patients with cystic neuroendocrine tumors and solid pseudopapillary neoplasms were excluded.

### 2.4. Development of the Convolutional Neural Network

**Figure 1:**
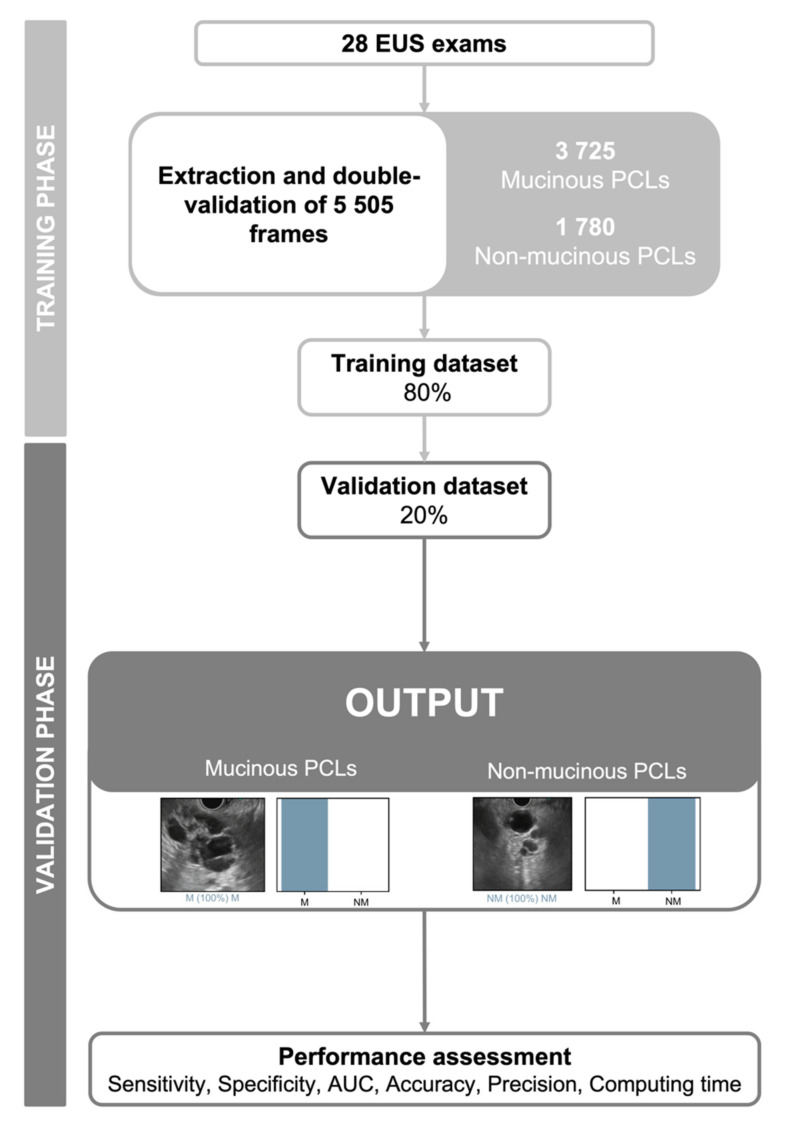
Study design for the construction of the convolutional neural network and subsequent evaluation of its performance. EUS—endoscopic ultrasound; PCLs—pancreatic cystic lesions; M—mucinous pancreatic cystic lesion; NM—non-mucinous pancreatic cystic lesion; AUC—area under the receiving operator curve.

A deep learning CNN was developed for the automatic identification and differentiation of mucinous and non-mucinous PCLs. In the former group, we included IPMNs and mucinous cystic neoplasms (MCN), while the latter included neoplastic (serous cystadenoma) and non-neoplastic (pseudocyst) lesions. From the collected pool of images (*n* = 5505), 3725 depicted mucinous and 1780 showed non-mucinous lesions. This pool of images was divided for the constitution of training and validation datasets. The training dataset was composed of 80% of the extracted images (*n* = 4404). The remaining 20% was used as the validation dataset (*n* = 1101). The performance of the CNN was assessed using the validation dataset. A flowchart summarizing the study design is presented in Figure 1.

The CNN was created using the *Xception* model with its weights trained on *ImageNet* (a large-scale image dataset aimed for use in development of object recognition software). To transfer this learning to our data, we kept the convolutional layers of the model. We removed the last fully connected layers and attached fully connected layers based on the number of classes we used to classify our endoscopic images. We used two blocks, each having a fully connected layer followed by a dropout layer of 0.25 drop rate. Following these two blocks, we add a dense layer with a size defined as the number of categories to classify (three: normal pancreatic parenchyma, mucinous PCLs and non-mucinous PCLs). The learning rate of 0.00015, batch size of 32, and the number of epochs of 30 was set by trial and error. We used *Tensorflow* 2.3 and *Keras* libraries to prepare the data and run the model. The analyses were performed with a computer equipped with a 2.1 GHz Intel^®^ Xeon^®^ Gold 6130 processor (Intel, Santa Clara, CA, USA) and a double NVIDIA Quadro^®^ RTX™ 4000 graphic processing unit (NVIDIA Corporate, Santa Clara, CA, USA).

### 2.5. Model Performance and Statistical Analysis

**Figure 2:**
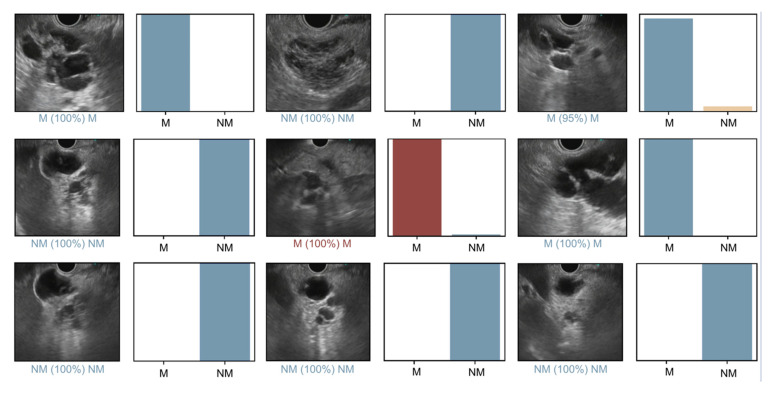
Output provided during the validation phase of the convolutional neural network. The bars represent the probability estimated by the algorithm. The finding with the highest probability was outputted as the predicted classification. A blue bar represents a correct prediction. Red bars represent an incorrect prediction. M—mucinous pancreatic cystic lesion; NM—non-mucinous pancreatic cystic lesion.

The primary outcome measures included sensitivity, specificity, positive and negative predictive values (PPV and NPV, respectively), and the accuracy in differentiating mucinous and non-mucinous lesions. Moreover, we used receiver operating characteristic (ROC) curves analysis and area under the ROC curves (AUC) to measure the performance of our model in the distinction between categories. The classification provided by the CNN was compared to the definitive diagnosis (mucinous or non-mucinous cyst), the latter being considered the gold standard. For each image, the trained CNN calculated the probability for each category. A higher probability translated in a greater confidence in the CNN prediction. The category with the highest probability score was outputted as the CNN’s predicted classification (Figure 2). Additionally, the image processing performance of the network was determined by calculating the time required for the CNN to provide output for all images in the validation image dataset. Clinical and demographic data are presented as median (interquartile range) or frequency (percent). Continuous data were compared using the Mann–Whitney U test. Differences in the distribution of categorical variables were assessed using the chi-square test. Statistical analysis was performed using Sci-Kit learn v0.22.2 [12].

## 3. Results

### 3.1. Clinical and Demographic Data

A total of 28 videos from patients submitted to EUS for pancreatic cystic lesion characterization between November 2017 and August 2021 were used for image retrieval. From these patients, 16 were female (57%) and had a median age of 65 years (IQR 53–70). A total of 17 (61%) individuals had a final diagnosis of mucinous cysts, while 11 (39%) were ultimately diagnosed with a non-mucinous lesion. Surgical specimens were reported for eight lesions. Histology using intracystic biopsy forceps samples (Moray^®^ micro forceps, STERIS) was available for five patients. The remaining cysts (*n* = 15) were considered mucinous based on fluid cyst analysis (cytology plus CEA and glucose levels). Concerning cyst histotype, we included 16 IPMNs, 1 mucinous cystic neoplasm (MCN), 8 SCA (five of which were of the macrocystic variant), and 3 pseudocysts (PC). The median follow-up time was 18 months (3–29). The characteristics of the patients and lesions including demographic data and lesion size and location are summarized in Table 1. Most lesions (86%) were incidentally found, 30% were located in the head and neck of the pancreas and the median size was 34.5 mm (19.3–44.8 mm). In this cohort, 14 patients underwent EUS for presumed mucinous lesions with worrisome features as per international consensus guidelines and 14 because of indeterminate cyst type after clinical and imaging integration (unilocular/oligocystic lesion without clear communication with the main pancreatic duct). Mucinous cysts were smaller in size compared to non-mucinous lesions, respectively, 26.0 mm (IQR 17.5–44.5) vs. 37.0 mm (IQR 26.0–46.0), although this difference was not statistically significant (*p* = 0.29). The location of the lesions had a similar distribution for mucinous and non-mucinous lesions (*p* = 0.90) and were more frequently found in the head and neck of the pancreas (47% and 55%, respectively). No adverse events were reported for the EUS procedures, nor for EUS-FNA (including through-the-needle biopsies).

**Figure 3:**
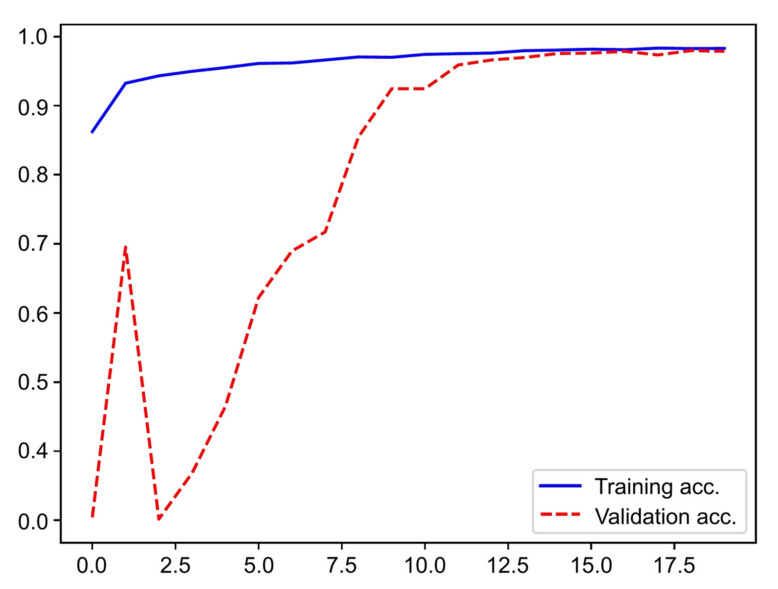
Evolution of the accuracy of the convolutional neural network during training and validation phases, as the training and validation datasets were repeatedly input in the neural network.

Overall, a total of 5505 frames were extracted for the construction of the CNN: 3725 of mucinous cysts (IPMNs and MCN) and 1780 of non-mucinous lesions (SCA and PC). The accuracy of the algorithm increased as data were repeatedly input into the multi-layer architecture of the CNN (Figure 3).

### 3.2. Performance of the Convolutional Neural Network

**Figure 4:**
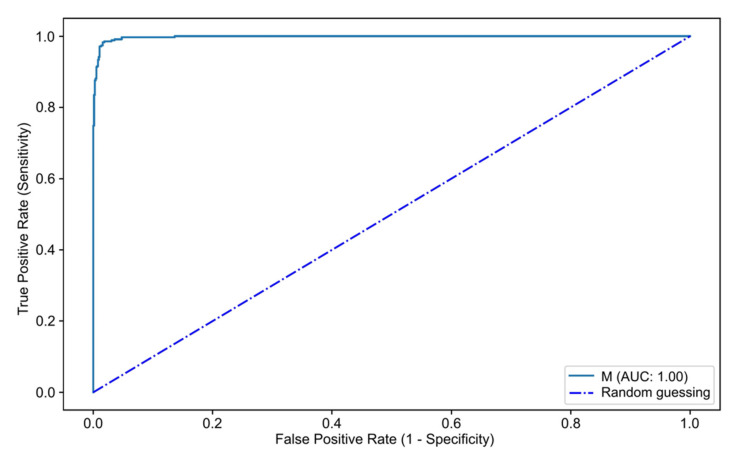
ROC analysis of the network’s performance in the detection of mucinous pancreatic cystic lesions. AUC—area under the receiver operating characteristic curve. M—mucinous pancreatic cystic lesion.

The full-size dataset was split for the constitution of training and validation datasets as follows: 80% of the retrieved images were used as a training dataset, and the remaining 20% were used as a validation dataset for evaluation of the CNN’s performance. The confusion matrix between the trained CNN and final diagnosis is shown in Table 2. Overall, the algorithm had an accuracy of 98.5%. The sensitivity, specificity, PPV and NPV for the detection and differentiation of mucinous cysts versus normal or non-mucinous structures were, respectively, 98.3%, 98.9%, 99.5% and 96.4%. The AUC of the CNN for the discrimination of mucinous and non-mucinous cystic lesions was 1.00 (Figure 4).

### 3.3. Computational Performance of the CNN

The CNN completed the reading of the validation dataset in 6 seconds at a speed of 5.2 ms/frame. This translates into an approximated reading rate of 191 frames per second.

## 4. Discussion

The development of AI algorithms is a hot topic in medical literature. Several reports show promising results regarding gains in diagnostic accuracy, particularly for medical specialties highly dependent on imaging [9]. The application of machine learning (ML) in endoscopy has shown encouraging results [10].

In this proof-of-concept study, we have developed a CNN for the automatic identification of mucinous pancreatic cysts during EUS. The algorithm demonstrated an excellent discriminatory ability with 98.5% accuracy for the differentiation of mucinous cysts from non-mucinous lesions. This proof-of-concept study represents a pilot effort to minimize the limited interobserver agreement regarding the EUS characterization of pancreatic cysts. Evidence on the application of AI to EUS for the study of pancreatic lesions is limited. Particularly, studies focusing on the detection and differentiation between mucinous and non-mucinous pancreatic cystic lesions based on EUS images are scarce. To the authors’ knowledge, only one study focusing on this subject has been previously published [13]. Nevertheless, the development of AI algorithms for the evaluation of pancreatic diseases is a subject of increasing interest [14,15,16,17,18,19].

The development of a deep learning model accurately predicting the phenotype of PCLs during EUS procedures may have a substantial impact on patient management. The main goals when approaching these lesions is defining their type (mucinous vs. non-mucinous) and, subsequently, attaining a definite histotype. The first step of this sequence is of particular relevance, as the malignant potential is virtually restricted to mucinous lesions. Therefore, we developed a deep learning algorithm for the automatic classification of PCLs as mucinous vs. non-mucinous. Nguon and coworkers implemented a CNN model to differentiate MCN and SCA using EUS images [13]. Their algorithm achieved an overall accuracy around 80%, which is in line with the classification performance of experienced endosonographers. The authors explained this suboptimal accuracy as the result of the inclusion of EUS images obtained using both radial and linear echoendoscopes as well as variations in the demarcation of single or multiple regions of interest (ROI), which included the cyst as well as surrounding tissue. This study differs from ours as we only included linear EUS images and our CNN model included complete images, without pre-selected ROI. Nevertheless, the main difference between the studies resides in the spectrum of included lesions, as our study focuses on group classification rather than differentiating between two different cyst types. Our model was built including EUS images from IPMNs in the mucinous group, in addition to MCN. IPMNs are the most frequent pancreatic cystic neoplasia and constitute a big challenge when it comes to correctly risk stratifying the malignant potential of each lesion. The work by Kuwahara et al. expands the reach of our study [15]. This group developed a deep learning algorithm to predict the malignancy potential of IPMNs using images from patients with malignant and non-malignant IPMNs. The authors used the output value of deep learning calculated after training as the predictive value of malignancy (AI value). The mean AI value of malignant lesions was higher than that of benign IPMNs. In this study, the CNN had a higher diagnostic performance than that of the endoscopists diagnosis and the predictive factors provided by scientific societies guidelines. Further studies on deep learning tools for application to EUS should expand the knowledge in this issue and address the challenge of automatic detection of cysts with advanced neoplasia, therefore minimizing the need for cyst puncture, ultimately preventing unnecessary surgeries.

The development of AI solutions for PCLs differentiation has expanded to other endoscopic tools complementary to conventional EUS. Confocal laser endomicroscopy (CLE) has been proven useful for differentiating various types of PCLs and more recently was shown to outperform international guidelines in the prediction of malignancy in IPMNs. However, image interpretation is observer dependent, and CLE is not widely available [20]. Recently, Machicado et al. described the development of a CNN algorithm based on CLE images to risk stratify IPMNs [16]. They used CLE videos from 35 histopathologically confirmed IPMNs and developed two CNN algorithms whose accuracy was compared to International Consensus Guidelines and American Gastroenterology Association criteria for advanced lesion/surgical indication. The results showed the higher accuracy of the CNN algorithm compared with the guidelines.

We conducted a proof-of-concept study assessing the potential of deep learning tools for the differentiation of mucinous and non-mucinous PCLs. This study has several highlights. First, to our knowledge, it is the first study to provide a clinically useful tool for the differentiation of PCLs as mucinous or non-mucinous. The accurate differentiation between both entities allows a prompt estimate of malignant potential, which has significant impact in patient management and follow-up. Second, our model was demonstrated to be highly sensitive, specific, and accurate. Finally, our algorithm had a high image processing performance with an approximate reading rate of 139 frames per second. An adequate image processing performance is a key element for subsequent real-time implementation of this proof-of-concept CNN model.

This study has several limitations. First, a small number of patients were enrolled and, therefore, some cyst types were underrepresented. The inclusion of a large pool of frames extracted from full-length videos, in addition to the routine still frames included in the standard EUS report, with distinct resolution and viewing angles contributed to provide an adequate variability to our dataset. Second, we performed a single-center retrospective study. Subsequent robust multicenter prospective studies are required to assess the clinical significance of our results. Further development of this technology will require the inclusion of large numbers of patients. Additionally, the refinement of the algorithm will require the inclusion of other types of pancreatic cysts as this should be required before it reaches clinical practice, providing automatic differentiation between several classes. Third, our proof-of-concept algorithm was developed and assessed using a single EUS suite. Therefore, our results may not be generalizable to other EUS platforms. Finally, the absence of surgical specimens or histological samples as the gold standard for all the included cysts is a significant limitation for establishing a reliable and reproducible gold standard for the development of the automated algorithm. An automated predictive model can only be as good as the gold standard for defining the true classification. Furthermore, the future application of AI tools into real-life EUS practice will require going through a strict regulatory pathway. The Food and Drug Administration (FDA) has approved several AI/ML-based Software as medical device (SaMD) with locked algorithms and changes beyond original market authorization requiring FDA premarket review [21]. Additionally, the FDA accepts the evolving and changing nature of AI/ML-based SaMD, namely convolutional neural networks. This particular matter constitutes a change from the previous paradigm for medical device regulation, as it was not initially designed for adaptive deep learning models. Indeed, a new framework is being gradually developed to provide appropriate regulatory oversight.

Artificial intelligence is gradually changing the landscape in digestive health care. Indeed, accurate, faster, and tireless AI tools will disrupt clinical practice and play a key role in endoscopic ultrasound. The potential of deep learning algorithms to impact the care of patients with pancreatic disease is vast and may contribute to improving the prognosis of these patients. We believe this AI model constitutes a significant milestone in the phenotypic differentiation of PCLs. Indeed, this work highlights the technological feasibility of accurately achieving morphologic pattern identification of pleomorphic pancreatic lesions. 

## Figures and Tables

**Table 1:** Baseline clinical and demographic data.

	Mucinous PCLs (*n* = 17)	Non-Mucinous PCLs (*n* = 11)	*p* Value
**Sex**			0.57
Female, *n* (%)	10 (58.8)	6 (54.5)	
**Age**			0.64
Years, median (IQR)	64.0 (53.0–69.5)	65.0 (53.0–72.0)	
**Presentation**			0.22
Incidental, *n* (%)	13 (76.5)	11 (100.0)	
Abdominal pain, *n* (%)	2 (11.8)	-	
Pancreatitis, *n* (%)	2 (11.8)	-	
**Indication for EUS**			<0.01
Worrisome features, *n* (%)	13 (76.5)	1 (9.1)	
Indeterminate cyst type, *n* (%)	4 (23.5)	10 (90.9)	
**Cyst location on EUS**			0.90
Pancreatic head, *n* (%)	8 (47.1)	6 (54.5)	
Pancreatic body, *n* (%)	6 (35.3)	3 (27.3)	
Pancreatic tail, *n* (%)	3 (17.6)	2 (18.2)	
**Cyst morphology**			0.63
Unilocular, *n* (%)	6 (35.3)	4 (36.4)	
Multilocular, *n* (%)	11 (64.7)	7 (63.6)	
**Cyst diameter**			
mm, median (IQR)	26.0 (17.5–44.5)	37.0 (26.0–46.0)	0.29

Abbreviations: EUS—endoscopic ultrasound; PCLs—pancreatic cystic lesions; IQR—interquartile range.

**Table 2:** Confusion matrix of the automatic detection versus final diagnosis.

		Final Diagnosis	
		Mucinous	Non-Mucinous
CNN	Mucinous	743	9
	Non-mucinous	12	337

Abbreviations: CNN—convolutional neural network; Mucinous—mucinous pancreatic cystic lesions; Non-mucinous—non-mucinous pancreatic cystic lesions.

## Data Availability

Data will be made available upon reasonable request.
